# Biventricular and Multisystem Thrombosis in a Young Patient With Elevated Factor VIII: A Case Report

**DOI:** 10.7759/cureus.92287

**Published:** 2025-09-14

**Authors:** Carl S Leib, Nicolás Ariza-Ordonez, Daniel Isaza

**Affiliations:** 1 Cardiology, Fundación Cardioinfantil, Bogotá, COL; 2 School of Medicine and Health Sciences, Universidad del Rosario, Bogotá, COL

**Keywords:** biventricular thrombosis, clinical case report, coagulation factor viii, hypercoagulable state, pulmonary embolism (pe), therapeutic anticoagulation, venous and arterial thrombosis, young adult thrombosis

## Abstract

Thrombosis in young adults without evident risk factors warrants investigation for underlying hypercoagulable states. We report the case of a 26-year-old male patient with a history of deep vein thrombosis (DVT), who presented with an extensive multisystem thrombotic cascade including acute limb arterial ischemia, bilateral pulmonary embolism, and bilateral renal vein thrombosis. Cardiac imaging revealed apical akinesis and a large biventricular thrombus with an ejection fraction of 41%. A comprehensive workup for thrombophilia identified a markedly elevated Factor VIII (FVIII) level as the only positive finding. This case underscores that high FVIII can drive a severe prothrombotic phenotype, leading to rare and devastating complications like biventricular thrombosis. The successful management with a multidisciplinary approach involving anticoagulation and antiplatelet therapy highlights the critical importance of early diagnosis to guide targeted therapy and prevent life-threatening recurrence.

## Introduction

Intracardiac thrombus formation, a serious and common cardiac event, is typically associated with conditions like atrial fibrillation, myocardial infarction, or heart failure. The occurrence of biventricular thrombosis is rare, particularly in young adults without overt risk factors, with only a limited number of case reports in the medical literature [1,2]. It usually arises in the presence of a hypercoagulable state, a pathological condition characterized by an increased tendency of blood to thrombose. Elevated levels of Factor VIII (FVIII), a cofactor for the proteolytic activation of Factor X by Factor IXa, are a recognized independent risk factor for venous thromboembolism (VTE) and arterial thrombosis. The pathophysiology involves an augmented coagulation response, which increases the propensity for thrombosis even in the absence of traditional risk factors such as smoking, hypertension, or hyperlipidemia. However, the association of a markedly elevated FVIII with a widespread, multisystemic thrombotic cascade that directly affects both sides of the heart is less common [3]. This report describes a young male patient with a prior deep vein thrombosis (DVT), who presented with biventricular and multisystem thrombosis associated with markedly elevated FVIII. The case emphasizes the necessity of considering hypercoagulable states, including FVIII elevation, in young patients with multiple thrombotic episodes. 

## Case presentation

A 26-year-old male patient with a history of DVT, previously untreated, presented with a two-week history of burning pain in the left lower limb, exacerbated by ambulation, and skin discoloration progressing from pallor to cyanosis. The patient reported no recent trauma, prolonged immobilization, or recent surgeries. He denied smoking, hypertension, or a family history of thrombotic disorders. On physical examination, the left lower limb was cold, mottled, and had absent distal pulses, consistent with arterial ischemia. Skin changes included cyanosis and edema. Vital signs were stable.

**Figure 1:**
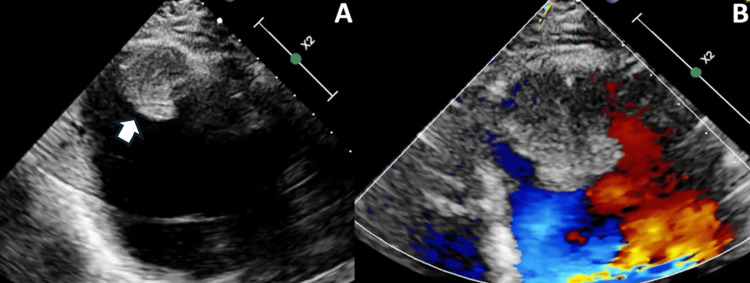
(A) Two-dimensional echocardiography shows an echodense, rounded mass adhered to the left ventricular apex (white arrow). (B) Apical zoom view color Doppler imaging reveals a lack of blood flow within the mass, confirming its solid, thrombotic nature.

Laboratory investigations revealed an elevated D-dimer of 3.2 μg/mL (reference range 0-0.4 μg/mL), a normal complete blood count, and normal liver and renal function tests. Contrast-enhanced CT angiography demonstrated occlusion of the left superficial femoral and popliteal arteries, bilateral pulmonary embolism involving multiple segments, and bilateral renal vein thrombosis. Echocardiography depicted anterior apical akinesis, hypokinesis of anterior and anterolateral ventricular walls, and a rounded, 34 x 23 mm echodense mass at the apex, suggestive of a thrombus. The left ventricular ejection fraction was 41% (Figure 1). 

**Figure 2:**
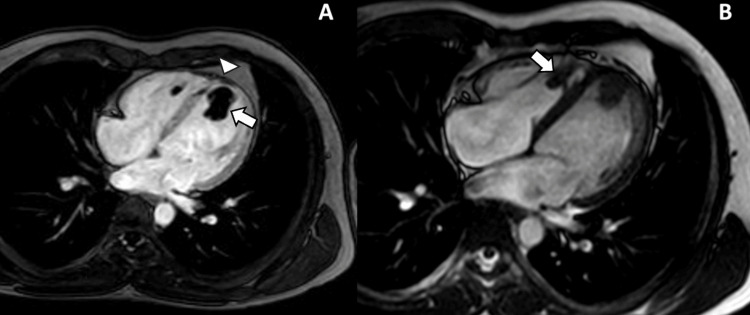
Cardiac MRI. (A) Phase-sensitive inversion recovery (PSIR) sequence demonstrates a hypointense signal at the left ventricular apex consistent with a thrombus (white arrow). The adjacent left ventricular myocardium shows region of infarction (white arrowhead). (B) A second hypointense thrombus is also visible at the right ventricular apex (white arrow).

Cardiac MRI confirmed a thrombus at the left ventricular apex, with infarction in the territories of the left anterior descending (LAD) and distal circumflex arteries. Additionally, it revealed a previously unrecognized 32 x 26 mm thrombus at the right ventricular apex (Figure 2).

Laboratory workup for hypercoagulability showed negative antiphospholipid antibodies, normal homocysteine levels, but markedly elevated FVIII activity at 460 IU/dL (reference range 50-150 IU/dL). Tests for additional hereditary thrombophilia, such as Factor V Leiden, prothrombin mutation, and protein C/S deficiencies, were negative. Screening for autoimmune conditions was also negative. There was no evidence for vasculitis or embolic disease from another source (Table 1). 

**Table 1:** Diagnostic findings summary CBC: Complete blood count; LV: Left ventricular; RV: Right ventricular; LAD: Left anterior descending; FVIII: Factor VIII

Category	Findings
Laboratory tests	D-dimer: 3.2 μg/mL (normal 0-0.4 μg/mL); CBC, liver, renal function: normal
CT angiography	Occlusion of left superficial femoral and popliteal arteries (arterial ischemia); Bilateral pulmonary embolism (multiple segments); Bilateral renal vein thrombosis
Echocardiography	Anterior apical akinesia; Hypokinesis of anterior and anterolateral ventricular walls; LV ejection fraction: 41%; LV apical thrombus: 34 × 23 mm echodense mass
Cardiac MRI	Confirmed LV apical thrombus; RV apical thrombus: 32 × 26 mm; Infarction in LAD and distal circumflex territories
Thrombophilia screen	FVIII activity: 460 IU/dL (normal 50-150 IU/dL); Antiphospholipid antibodies: negative; Homocysteine: normal; Factor V Leiden mutation: negative; Protein C/S deficiency: negative; Autoimmune thrombophilia: negative

The patient was initiated on warfarin with bridging low-molecular-weight heparin, and single antiplatelet therapy was added due to arterial involvement. The multidisciplinary team decided on outpatient follow-up, with plans for repeat echocardiography at three months to monitor thrombus resolution. During hospitalization, supportive measures included limb revascularization procedures, pain management, and close monitoring for complications. The elevated FVIII was interpreted as a primary hypercoagulable state contributing to the extensive thrombotic presentation.

At the three-month follow-up, a repeat echocardiogram revealed partial resolution of the biventricular thrombosis. The patient reported clinical improvement, and there was no evidence of new thrombotic episodes. Warfarin therapy was continued, with plans for long-term anticoagulation, considering the persistently elevated FVIII.

## Discussion

This case details the complex and challenging presentation of a young male adult with multisystem and biventricular thrombosis attributed to a previously undiagnosed hypercoagulable state. The confluence of extensive VTE, arterial ischemia, and intracardiac thrombi in a patient with no conventional risk factors is highly unusual and prompted an extensive workup for thrombophilia [4]. Our findings confirm that markedly elevated FVIII levels, at 460 IU/dL, were a critical etiological factor in this patient's severe thrombotic phenotype. Recent research continues to support that elevated FVIII is an independent risk factor for thrombosis, with studies reinforcing its importance in the etiology of venous and even cerebral venous thrombosis [3,5].

Elevated FVIII levels have been increasingly recognized as a significant, independent risk factor for VTE, with prospective studies demonstrating a dose-dependent relationship between FVIII activity and thrombotic risk [6]. While this association is well-established for VTE, our case highlights its profound contribution to both arterial and intracardiac thrombosis, a less common manifestation. The presence of bilateral renal vein thrombosis, pulmonary embolism, and limb arterial ischemia in our patient suggests an overwhelming prothrombotic environment affecting both the venous and arterial circulations [7].

A defining feature of this case is the development of biventricular thrombosis, a rare finding. The mechanism likely involves a combination of the systemic hypercoagulable state and localized factors, such as the apical akinesis observed on echocardiography. Regional wall motion abnormalities can cause blood stasis, creating an environment prone to thrombus formation in the presence of elevated FVIII levels. The finding of a thrombus on cardiac MRI, alongside infarction in the LAD and circumflex territories, further suggests that the cardiac events were likely thrombotic in origin, possibly from in-situ thrombosis or microembolization, rather than traditional atherosclerotic disease [8,9].

Management of this patient was complex and required a multidisciplinary approach. Recent case reports have highlighted the successful treatment of large biventricular thrombi with medical therapy, even in the context of other comorbidities, reinforcing the role of aggressive anticoagulation in these severe cases [10]. The treatment with oral anticoagulation (warfarin) and antiplatelet therapy (aspirin) was initiated to target both venous and arterial thromboses. Long-term anticoagulation is crucial for patients with unprovoked thrombosis and persistently elevated FVIII, given the high risk of recurrence. The successful partial resolution of the biventricular thrombi and the absence of new thrombotic episodes at the three-month follow-up underscore the effectiveness of this tailored management strategy [3]. This case serves as an important reminder to screen for rare thrombophilias, such as elevated FVIII, in young patients with atypical or extensive thrombotic events, as early diagnosis and appropriate management are paramount to improving outcomes.

This case report is limited by the absence of long-term follow-up beyond three months, which precludes conclusions regarding the durability of thrombus resolution and the risk of recurrent thrombosis over time. As a single case, the findings cannot be generalized, but they highlight the need for larger studies to better understand the clinical spectrum and management of FVIII thrombophilia.

## Conclusions

This case describes a rare presentation of biventricular and multisystem thrombosis in a young male patient, with markedly elevated FVIII identified as the underlying cause. The confluence of extensive venous, arterial, and intracardiac thrombi in a patient without conventional risk factors highlights the importance of investigating for hypercoagulable states. The partial resolution of the thrombi and the absence of new events under targeted anticoagulation emphasize the value of maintaining a high index of suspicion, ensuring timely diagnosis, and implementing aggressive management. Furthermore, this case illustrates how recognizing uncommon risk factors such as elevated FVIII may inform future diagnostic strategies and therapeutic approaches in young patients presenting with unexplained or extensive thrombosis. 
